# Antidepressant treatment in premenstrual dysphoric disorder, case report

**DOI:** 10.1192/j.eurpsy.2022.841

**Published:** 2022-09-01

**Authors:** M. Garcia Moreno, A. De Cos Milas, L. Beatobe Carreño, M.B. Poza Cano, A. Izquierdo De La Puente, P. Del Sol Calderón

**Affiliations:** 1 HOSPITAL UNIVERSITARIO PUERTA DE HIERRO MAJADAHONDA, Psychiatry, MADRID, Spain; 2 HOSPITAL UNIVERSITARIO DE MÓSTOLES, Psychiatry, MADRID, Spain; 3 HOSPITAL UNIVERSITARIO EL ESCORIAL, Psychiatry, MADRID, Spain; 4 Hospital Universitario Puerta de Hierro, Psiquiatría Infanto-juvenil, Madrid, Spain

**Keywords:** depressive disorder, premenstrual, women, Treatment

## Abstract

**Introduction:**

Premenstrual dysphoric disorder (PMDD) is included for the first time in the last edition of DSM within affective disorders. It is necessary that 5 of a list of 11 symptoms (lability, irritability, depressed mood, anxiety, lethargy, being out of control or physical symptoms among others) appear in the majority of menstrual cycles but must be only present during the week before menstruation improving after its onset. It has a prevalence of 1,8-5,8% and it is associated to significant functional impairment. SSRIs are indicated as first-line treatment in severe symptoms.

**Objectives:**

To review about premenstrual dysphoric disorder and its psychopharmacological treatment.

**Methods:**

We carry out a literature review about premenstrual dysphoric disorder, accompanied by a clinical description of one patient treated with sertraline.

**Results:**

44 years old female referred to our outpatient mental health service due to anxious and depressive symptoms. She had presented abdominal pain, anxiety, obsessive thoughts, sadness, emotional lability, apahty, anergy, uncontrolled impulse, irritability and vociferous attitude with verbal agressiveness only the week before menstruation during several years. These symptoms interfered negatively in her relationships. We started sertraline treatment with ad integrum clinical recovery after two menstrual cycles. 6 months later we indicated to take sertraline only the week before menstruation, maintaining stability.

**Conclusions:**

1) It is important to consider premenstrual dysphoric disorder as a possible diagnosis in women with premenstrual discomfort symptoms. 2) It might be consider as a depressive disorder. 3) Antidepressant treatment should be considered in women with disabling symptoms.

**Disclosure:**

No significant relationships.

